# Brd2 Inhibits Adipogenesis via the ERK1/2 Signaling Pathway in 3T3-L1 Adipocytes

**DOI:** 10.1371/journal.pone.0078536

**Published:** 2013-10-23

**Authors:** Kun Zang, Jingyu Wang, Miaofang Dong, Ruixin Sun, Yuxiong Wang, Yinong Huang, Xiaoxia Liu, Yimin Li, Fangnian Wang, Min Yu

**Affiliations:** 1 The Key Laboratory of Molecular Medicine, Ministry of Education, Fudan University, Shanghai, PR China; 2 Department of Biochemistry and Molecular Biology, Shanghai Medical College, Fudan University, Shanghai, PR China; 3 Department of Endocrinology, Hua Shan Hospital, Fudan University, Shanghai, PR China; Wayne State University, United States of America

## Abstract

Bromodomain-containing protein 2 (Brd2) is a nuclear serine/threonine kinase involved in transcriptional regulation. In 3T3-L1 adipocytes, Brd2 normally co-represses PPARγ (peroxisome proliferator-activated receptor gamma) and inhibits adipogenesis. Here, we show that Brd2 over-expression in preadipocytes inhibits their differentiation into adipocytes, while Brd2 knockdown promotes adipogenic differentiation *in vitro* and forces cells to undergo adipogenesis independent of the MDI (methyisobutylxanthane, dexamethasone and insulin) induction. In this study, the two key transcription factors for adipogenesis, PPARγ and C/EBPα (CCAAT/enhancer binding protein-α) were persistently expressed during the differentiation of preadipocytes to mature adipocytes in Brd2 knockdown 3T3-L1 cells, but their expression was inhibited in cells in which Brd2 was overexpressed. To investigate the role of Brd2 in signal transduction we examined the expression of several signaling molecules involved in the regulation of gene expression and cell differentiation by immunoblotting assay. Down-regulation of Brd2 expression in 3T3-L1 cells led to a decrease in extracellular signal-regulated kinase1/2 (ERK1/2) activity and, conversely, the up-regulation of Brd2 leads to increase in ERK1/2 phosphorylation. Nevertheless, changes in Brd2 expression do not affect the activities of JNK and p38 MAPK. In addition, the phosphorylation of Rafs is not affected by changes in Brd2 expression in 3T3-L1 cells. MEK inhibitor UO126 partly restores differentiation of 3T3-L1 cells that overexpress Brd2. In conclusion, these results indicate that Brd2 regulates ERK1/2 activity independently of Raf signaling in 3T3-L1 adipocytes.

## Introduction

Obesity is one of the most frequent physiological disorders that is associated with a wide variety of conditions including hypertension [1], dyslipidemia [2], atherosclerosis [3], type II diabetes [4], nonalcoholic fatty liver disease [5], periodontal disease [6], asthma [7], cardiovascular diseases [8] and certain cancers[Dalla Vecchia, 2005 #19678] [9,10]. In addition, the number of obese individuals is continuously increasing and is becoming a serious health problem worldwide [11-13]. 

Adipocytes are the major cellular components of fat tissue and excessive growth, differentiation and hypertrophy of adipocytes are fundamental processes of obesity. Maturation of adipocytes can occur among cells from a pre-existing pool of adipocyte progenitor cells (preadipocytes) and be present irrespective of age [14]. In the adipocyte differentiation process, PPARγ (peroxisome proliferator-activated receptor gamma) and C/EPBα (CCAAT enhancer-binding protein-α) play central roles by regulating expression of adipocyte-specific genes [15], especially. The function of PPARγ is particularly important and probably sufficient for adipogenesis. PPARγ can be activated by fatty acid derivatives or antidiabetic drugs (e.g. thiazolidinediones), while posttranslational modifications such as phosphorylation can decrease PPARγ activity [16]. 

Mitogen-activated protein kinases (MAPKs), including extracellular regulated protein kinases (ERK), c-Jun N-terminal kinase (JNK) and p38 MAPK, are kinases involved in PPARγ phosphorylation and inhibition of its activity [17,18]. The MAPK/ERK pathway regulates numerous cellular processes in normal cells as well as in tumor cells, but its main function lies in the control of cell survival and proliferation [19]. The MAPK/ERK signaling cascade is organized hierarchically into a three-tiered module composed of MAPKKK (Raf), MAPKK (MEK1/2) and MAPK (ERK1/2). Activation and inactivation of the Raf/MEK/ERK signal transduction cascade is tightly regulated by protein kinases as well as protein phosphatases [20,21]. The role of JNK in the pathogenesis of obesity, insulin resistance, and type 2 diabetes was first suggested by the demonstration that deletion of the JNK1 gene (JNK12/2) leads to decreased adiposity and significant improvements in insulin sensitivity in both dietary and genetic (ob/ob) mouse models of obesity [22]. p38 MAPK is active in 3T3-L1 fibroblasts and developing adipocytes, and its activity decreases dramatically during later stages of differentiation [23].

Bromodomain-containing protein 2 (Brd2) is a nuclear transcription factor kinase that acts as a co-regulator in the regulation of gene transcription [24]. Previous studies have shown that Brd2 can bind transcriptional activators like E2F proteins, and coactivator TATA binding factor (TBP)-associated factors (TAFs), members of the SWItch/sucrose nonfermentable (SWI/SNF) complex, histone acetyltransferases (HATs) and histone deacetylases (HDACs) to regulate the expression of diverse genes [25]. A nuclear/cytoplasmic translocation of Brd2 protein has been observed in cultured mouse fibroblasts as well as in mammary epithelial cells during the reproductive cycle, correlating with both proliferation and apoptosis [26]. In addition, a recent study demonstrated that Brd2 could co-repress PPARγ and thus inhibits adipogenesis [27]. Furthermore, disruption of Brd2 in mice causes severe obesity without type 2 diabetes [27]. 

Although the inhibitory role of Brd2 in adipocyte differentiation has been well established, signaling pathways downstream of Brd2 have not been elucidated until recently. In vitro assays demonstrate that ERK is able to phosphorylate PPARγ, resulting in a decrease of PPARγ transcriptional activity [16]. In this study we have examined whether Brd2-inhibited adipogenesis is associated with ERK activation in 3T3-L1 preadipocytes.

## Results

### 1: Characteristics of transfected 3T3-L1 cells

**Figure 1:**
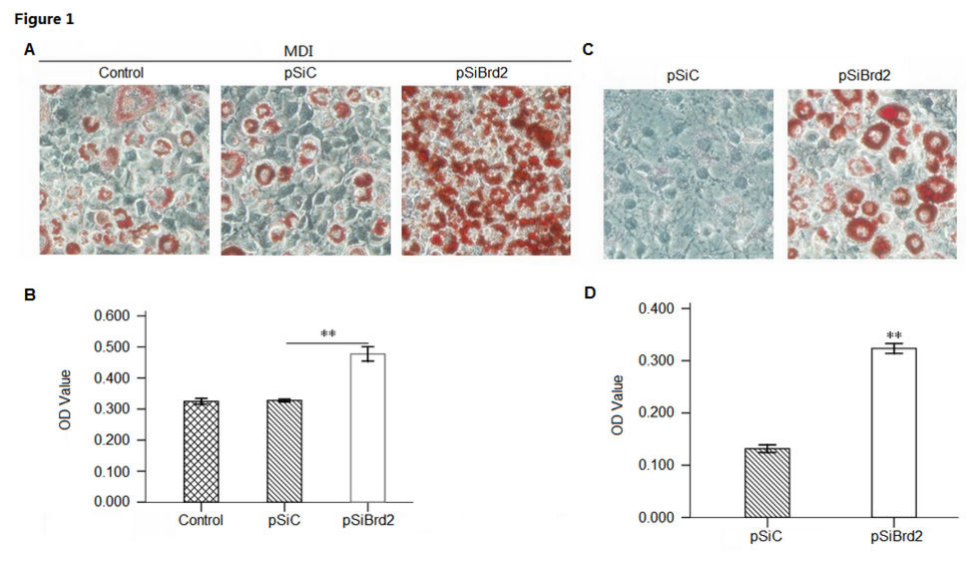
Characteristics of 3T3-L1 cells with Brd2 knockdown. 3T3-L1 cells were transfected with Brd2 shRNA (SiBrd2) or scramble shRNA (SiC) plasmids respectively. (A) Adipogenic differentiation of 3T3-L1 adipocytes from pre-adipocytes and Oil Red O staining on day 4 of treatment reveals lipids in cells that have undergone differentiation. (B) Quantification of lipid content in transfected 3T3-L1 cells after adipogenic differentiation (n = 3; **P < 0.01). (C) 3T3-L1 cells transfected with Brd2 shRNA (SiBrd2) or scramble shRNA (SiC) plasmids, respectively, cultured without MDI induction for 8 days, and then stained with Oil Red O. (D) Quantification of lipid content in transfected 3T3-L1 cells after adipogenic differentiation (n = 3; **P < 0.01).

**Figure 2:**
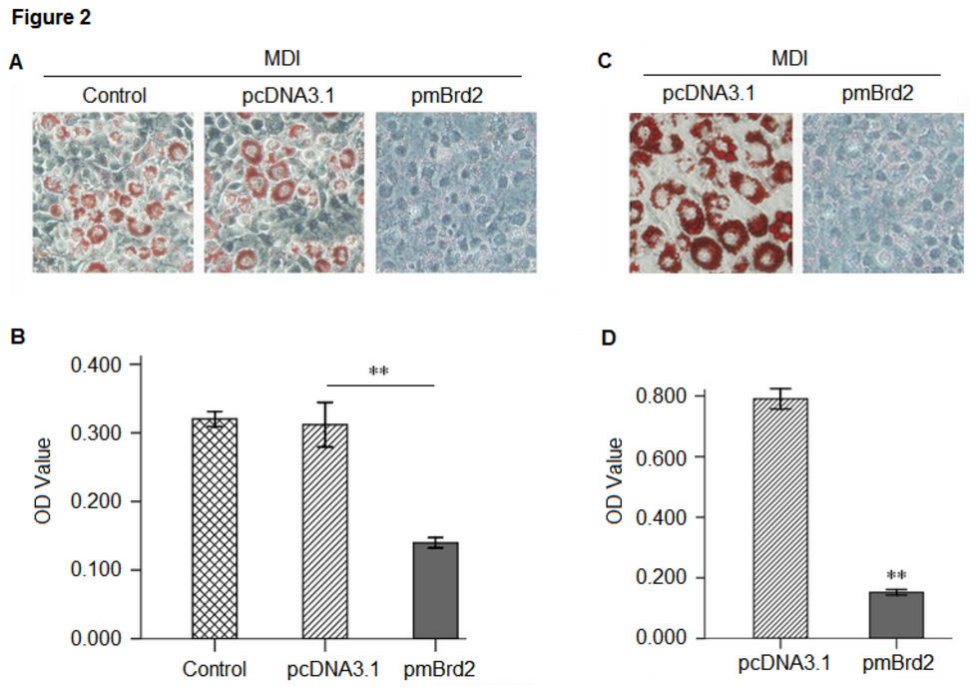
Characteristics of 3T3-L1 cells with overexpressed Brd2. 3T3-L1 cells transfected with pmBrd2 and control (pcDNA3.1), respectively. (A) Adipogenic differentiation of 3T3-L1 adipocytes from pre-adipocytes and Oil Red O staining on day 4 of treatment reveals lipids in cells that have undergone differentiation. (B) Quantification of lipid content in transfected 3T3-L1 cells after adipogenic differentiation (n = 3; **P < 0.01). (C) 3T3-L1 cells transfected with pmBrd2 and pcDNA3.1 respectively induced with MDI, and on day 8, stained with Oil Red O. (D) Quantification of lipid content in transfected 3T3-L1 cells after adipogenic differentiation (n = 3; **P < 0.01).

In order to examine whether Brd2 might alter the differentiation program of the preadipocytic cell line, 3T3-L1 cells were transfected with plasmids (pSiBrd2, pmBrd2 and controls) and then induced to differentiate. On days 4 and 8 of treatment, differentiation was assessed by staining lipid droplets with Oil red O. Control cells (transfected with pSiC and pcDNA3.1) that differentiated into fat cells and accumulated lipids were stained. The more intensive staining was present in Brd2 knockdown cells (Figure 1) while the less intensive staining was observed in cells with Brd2 overexpression (Figure 2). This indicates that Brd2 knockdown could possibly promote adipogenic differentiation and this promotion might force cells to undergo adipogenesis without MDI induction, as shown qualitatively and quantitatively in Figure 1C and D. Conversely, overexpression of Brd2 strongly suppressed adipogenic differentiation (Figure 2C and D).

### 2: Expression of PPARγ and C/EBPα in 3T3-L1 adipocytes

**Figure 3:**
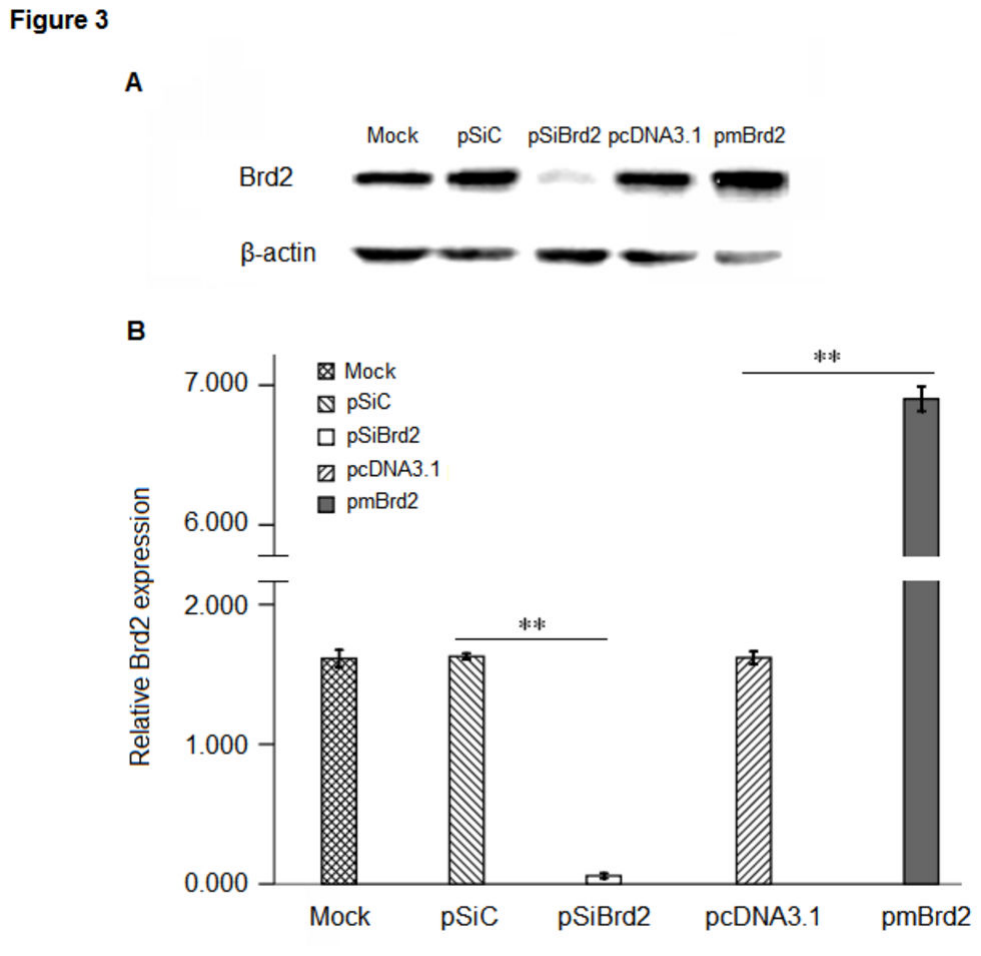
Effective inhibition and increased Brd2 expression in 3T3-L1 adipocytes. (A) WT 3T3-L1 adipocytes were transfected with the Brd2 shRNA (SiBrd2), scramble shRNA (SiC) plasmids, pmBrd2 and control (pcDNA3.1), respectively. After transfection (48 h), cells were passaged, and treated with selection of antibiotics G418 for stable transfection. Single colonies were expanded and cells were harvested to examine Brd2 expression by immunoblotting with anti-Brd2 antibody. (B) Brd2 expression levels were quantified by densitometry in arbitrary units (AU) using ImageJ software and normalized to the amount of β-actin. Data are expressed as mean ± SEM of at least three experiments. **P < 0.01 by Student *t*-test.

Adipogenesis, the process by which adipocyte precursors develop into adipocytes, is regulated by a group of specific transcription factors, such as PPARγ and C/EPBα. They play critical roles in this process and their functions are necessary and probably sufficient for adipogenesis. Therefore we wanted to examine whether the levels of PPARγ and C/EPBα change after knockdown or overexpression of Brd2 in 3T3-L1 cells (Figure 3). 

**Figure 4:**
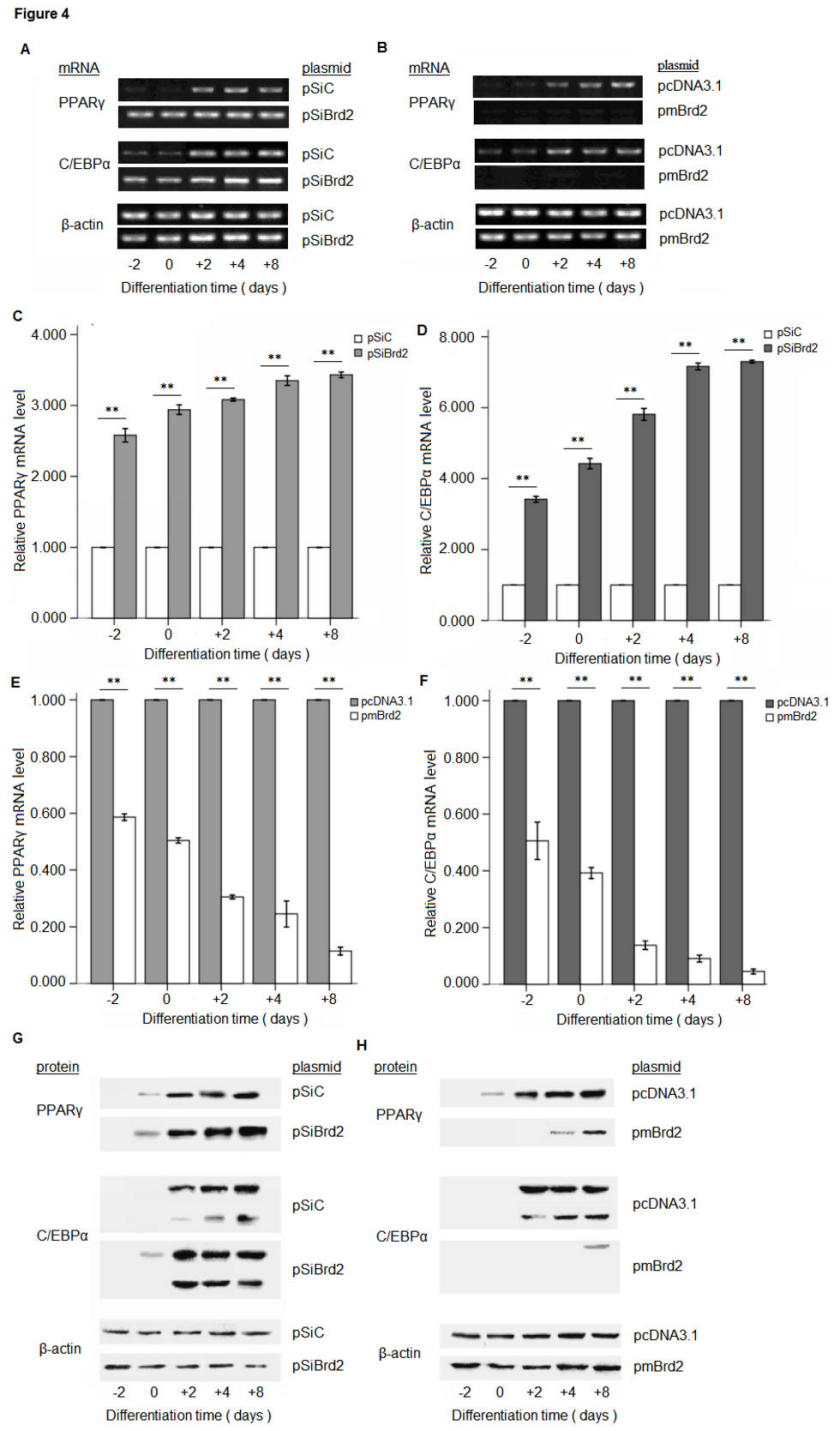
Analysis of Brd2 influence on PPARγ and C/EBPα expression. (A) PPARγ and C/EBPα mRNA expression in Brd2 knockdown 3T3-L1 cells, and (B) in Brd2 overexpression 3T3-L1 cells, as detected by reverse transcription PCR at different time points during adipogenic differentiation. mRNA expression was analyzed using reverse transcription PCR. (C and D) Quantitative real-time PCR expression analysis of PPARγ and C/EBPα in 3T3-L1 cells with Brd2 shRNA, and (E&F) with pmBrd2, relative mRNA levels were determined by △△Ct =△Ct, _sample_ —△Ct, _reference_ and β-actin was used as the reference gene. (n = 3, **P < 0.01). (G) The protein levels of PPARγ and C/EBPα in 3T3-L1 cells, which were transfected with pSiBrd2 or pSiC respectively, and (H) in 3T3-L1 cells which were transfected with pmBrd2 and control (pcDNA3.1) by western blotting at different time points during adipogenic differentiation. Each blot is representative of those obtained from triplicate experiments.

We have induced differentiation of these cells and isolated total RNA at five different time points, day -2 and day 0 (uninduced cells), day +2 and day +4 (induced cells), and day +8 (terminally differentiated cells). PPARγ and C/EPBα mRNA expression was analyzed by quantitative RT-PCR. In the control experiments, we confirmed that PPARγ and C/EPBα mRNA expression was observed after induction, but not before the differentiation of 3T3-L1 adipocytes. When Brd2 was knocked down, both PPARγ and C/EPBα levels increased immediately, and remained high until the terminal differentiation, while Brd2 overexpression inhibited the induction of C/EPBα and PPARγ (Figure 4). These results suggest that Brd2 may inhibit adipogenic differentiation by suppressing the PPARγ and C/EPBα expression. Consistent with this, endogenous Brd2 expression in 3T3-L1 adipocytes declines during adipogenic differentiation (Figure S1).

### 3: MAPKs activities in transfected cells

**Figure 5:**
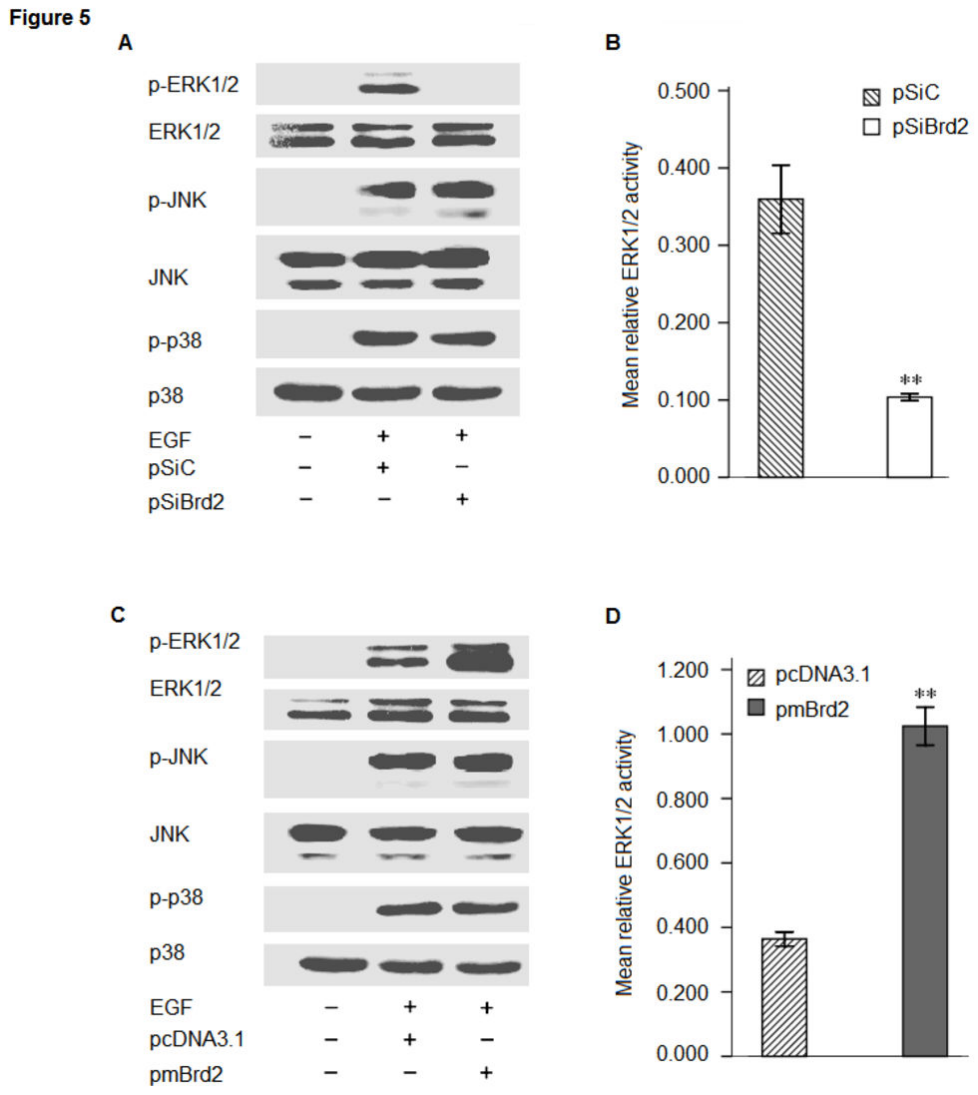
Activation of MAPKs in transfected 3T3-L1 adipocytes. Western blotting was performed with phosphorylated ERK1/2 (Thr202/Tyr204) and total ERK1/2 antibodies, anti-phosphop-JNK at Thr183/Tyr185 and total JNK, anti-phosphop-p38 MAPK at Thr180/Tyr182 and total p38 MAPK antibodies. (A) 3T3-L1 cells were transfected with Brd2 shRNA (SiBrd2) or control (SiC) plasmids, and (B) ERK1/2 phosphorylation was quantified by densitometry in arbitrary units (AU) using ImageJ software, normalized to total amount of ERK1/2. (C) 3T3-L1 cells were transfected with pmBrd2 or control, and (D) ERK1/2 phosphorylation was quantified using the same method as in (B). Each blot is representative of those obtained from triplicate experiments; data are expressed as mean ± SEM. **P < 0.01, Student *t*-test.

To assess the degree of ERK1/2 activation, a monoclonal antibody recognizing dually phosphorylated ERK1/2 (Thr202/Tyr204) was employed on whole cell lysates from pSiBrd2, pmBrd2 and control transfected cells. We found that Brd2 knockdown strongly suppressed the phosphorylation of ERK1/2 (Figure 5A and B); conversely, an increase in activated Erk1/2 was observed in pmBrd2 cell lysates compared with cell lysates from pcDNA3.1 cells (Figure 5C and D). JNK and p38 MAPK were not involved in the process of Brd2 regulated adipogenesis (Figure 5A and C).

### 4: Brd2 regulated ERK1/2 activity in a Raf-independent manner

**Figure 6:**
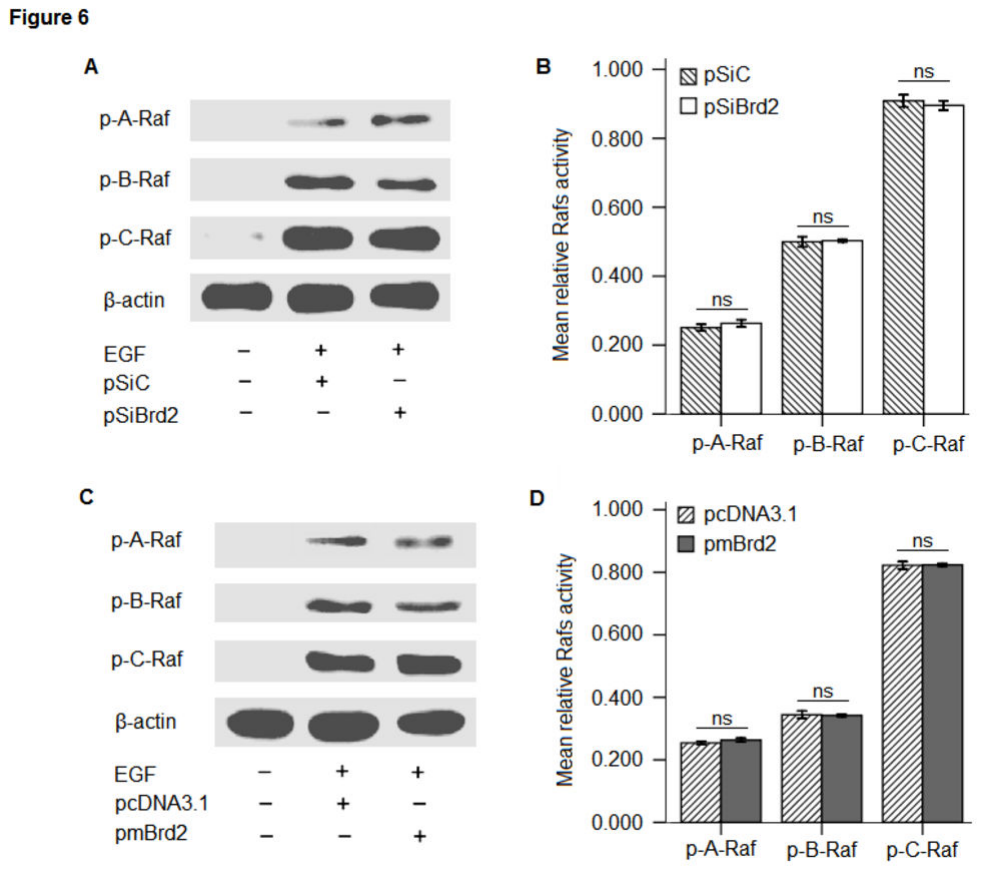
Brd2 activates ERK1/2 in a Raf-independent manner. Expression of phosphorylated A-Raf (Ser299), phosphorylated B-Raf (Ser445), and phosphorylated C-Raf (Ser338) was examined in protein lysates isolated from transfected cells. (A) Indicative blot representing the expression of target protein after transfection with pSiBrd2 or pSiC. (B) Raf phosphorylation was quantified by densitometry in arbitrary units (AU) using ImageJ software, normalized to β-actin. (C) Indicative blot representing the expression of target proteins after transfection with pmBrd2 or pcDNA3.1. Western blotting was performed with a Raf Family Antibody Sampler Kit. (D) Rafs phosphorylation was quantified using the same method as (B). Each blot is representative of those obtained from triplicate experiments, data are expressed as mean ± SEM. ns, not significant as determined by Student *t* test compared to controls.

In order to examine how ERK1/2 activity is regulated by Brd2 in 3T3-L1 preadipocytes, the activation state of the upstream MEK regulators, the Raf family members, was evaluated in cells with Brd2 silencing or overexpression. The phosphorylation state of Raf family was determined by Western blot analysis using phospho-specific antibodies that specifically detect the activated form of each kinase. In contrast to ERK1/2 activation, neither A-Raf, B-Raf nor C-Raf were significantly hyperphosphorylated in an activating manner by Brd2 overexpression, as opposed to their status in cells with Brd2 silencing. These data suggest that the Raf family phosphorylation/activation status is not associated with Brd2, possibly because Brd2 may act downstream of Raf, with no feedback regulation to Raf in 3T3-L1 preadipocytes. However, contrary to activatory effects on ERK1/2 kinase activity, Brd2 had no apparent effect on the activation of Rafs (Figure 6).

### 5: MEK inhibitor partly restores differentiation of transfected 3T3-L1 cells

**Figure 7:**
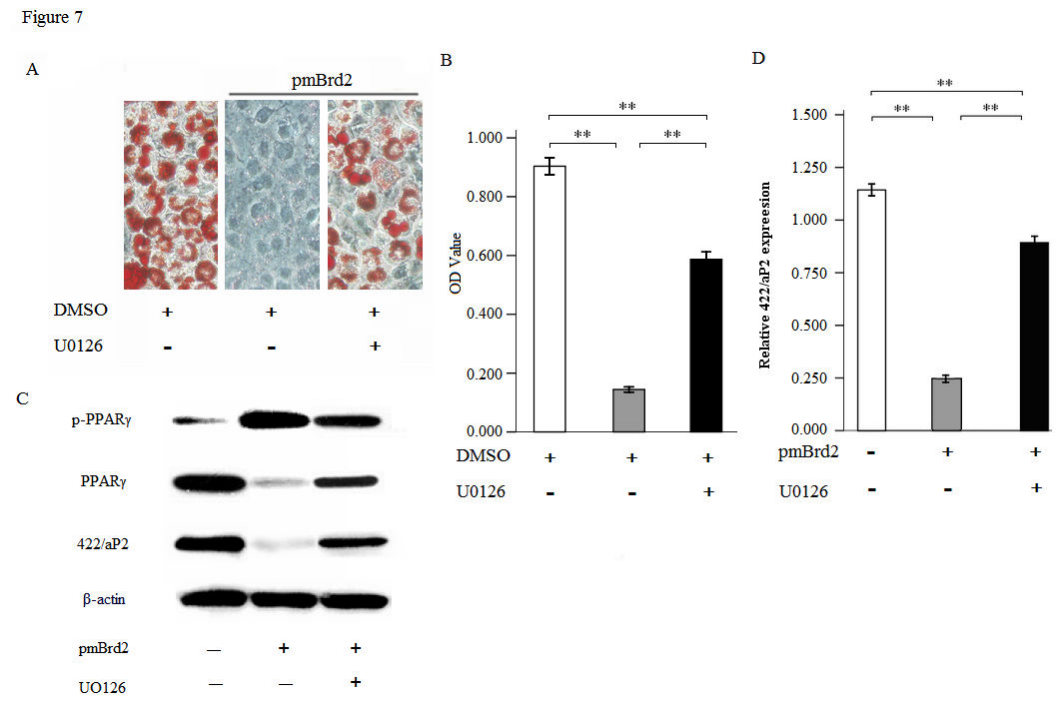
Inhibition of MEK signaling leads to the resaturation of differentiation in cells with Brd2 overexpression. 3T3-L1 cells transfected with pmBrd2 were induced to differentiate in the presence of UO126 or dimethylsulfoxide (DMSO) as a vehicle, and WT 3T3-L1 as a control. (A) Adipogenic differentiation of 3T3-L1 adipocytes from pre-adipocytes was performed and on day +8, cells were fixed, and then stained with Oil Red O. (B) Quantification of lipid content in transfected 3T3-L1 cells after adipogenic differentiation. (C) Adipogenic differentiation of 3T3-L1 adipocytes from pre-adipocytes was performed and on day +8, Western blotting was performed with p-PPARγ, PPARγ, 422/aP2 and β-actin antibodies. (D) 422/aP2 expression levels were quantified by densitometry in arbitrary units (AU) using ImageJ software, normalized to the amount of β-actin. All data are expressed as mean ± SEM and representative results of at least three experiments are shown. **P < 0.01, Student *t*-test.

If excessive ERK1/2 signaling is responsible for the effect of differentiation by Brd2, then inhibition of ERK1/2 signaling should restore the differentiation potential of pmBrd2 transfected 3T3-L1 cells. In order to examine this hypothesis we induced adipocyte differentiation in the presence of the MEK inhibitor UO126 that blocks the phosphorylation and activity of ERK1/2. When pmBrd2 transfected 3T3-L1 cells were induced to differentiate in the presence of UO126, they were able to regularly differentiate into adipocytes whereas vehicle-treated (dimethylsulfoxide, DMSO) cells remained resistant to differentiation (Figure 7A and B). PPARγ is also a phosphoprotein phosphorylated by activators of MAPK, like insulin; however, this modification decreases its transcriptional activity [17]. In addition, the treatment of cells with MEK kinase inhibitor inhibits the degradation of PPARγ [28]. Thus when UO126 was used to treat cells that overexpressed Brd2, the phosphorylated PPARγ decreased, and the degradation of PPARγ was inhibited. In addition, 422/aP2, a marker of adipogenesis also showed partial recovery (Figure 7C and D). These results therefore suggest that activation of the Erk1/2 was involved in the pathway leading to inhibition of adipogenesis by Brd2.

## Discussion

Brd2, formerly ‘RING3’, is a member of the bromodomain and extraterminal domain (BET) subfamily of proteins that harbours two bromodomains [29,30]. The zebrafish homologue of the Brd2 gene is highly expressed in the egg, early embryo, and during nervous system development [31]. Various functions have been ascribed to the Brd2 protein, including transactivation of promoters of several cell cycle regulatory genes [32], binding to mitotic chromosomes [33], and interaction with acetylated lysine-12 in histones [34]. Although changes in expression of Brd2 have been linked to certain human cancers, the newly described bromodomain protein Brd2 also performs profound, diverse and non-redundant functions in adipogenesis, energy metabolism and inflammation [35]. Adipogenesis is crucial for energy balance, and its deregulation can cause lipoatrophy or obesity. In this processes, a group of transcription factors, such as PPARγ and C/EPBα, play critical roles and their functions are necessary and probably sufficient for adipogenesis [36,37]. *In vitro* studies have shown that Brd2 binds to PPARγ and inhibits its transcriptional activity [27]. This in turn leads to the suppression of PPARγ induced C/EPBα expression, and due to the positive feedback loop between C/EBPα and PPARγ [37], their expression was low when Brd2 was overexpressed (Figure 4). This may explain the decreased adipogenic differentiation in 3T3-L1 cells with overexpression of Brd2 (Figure 3). 

While Brd2 was defined as a member of a new class of kinases [24], the mechanisms accounting for its involvement in signal transduction and kinase cascade activation still remain unknown. Denis et al. (1998) demonstrated that RING3 kinase could be activated by growth factors such as EGF in the lung and brain. However, no attempt was made to correlate increases in ERK activity with the induction of Brd2 in adipocytes. The results presented here show for the first time that Brd2 is capable of increasing the ERK activation in 3T3-L1 cells (Figure 5). Interestingly, unlike previous reports demonstrating direct activation of ERK activity by Raf [38,39], Brd2 activated ERK1/2 in a Raf-independent manner (Figure 6). Activation of MAPK had profound effects on the differentiation program of 3T3-L1 preadipocytes by increasing the phosphorylation of PPARγ [17]. EGF stimulated MEK-dependent phosphorylation, and co-transfection of adipocytes with a constitutively active MEK resulted in a decrease in PPARγ transcriptional activity. In vitro assays have demonstrated that ERK2 and JNK are able to phosphorylate PPARγ2 [18]. Conversely, treatment of cells with an inhibitor of MEK kinases inhibits the degradation of PPARγ, and can be sufficient to restore the adipogenic differentiation program [40]. Since UO126, the MEK inhibitor, could apparently abolish Brd2-induced inhibition of differentiation in 3T3-L1 preadipocytes (Figure 7A and B), the interaction between Brd2 and ERK activation might occur at the level of MEK1/2. This is further supported by the differentiation recovery in 3T3-L1 cells with Brd2 overexpression after the UO126 treatment. How the interaction between Brd2 and MEK occurs remains unknown. Multiple MEK kinases (MEKK), in addition to Raf, leading to the activation of ERK have been identified, including MEKK1, -2, -3, and -4 [41]. It is therefore possible that a MEK kinase other than Raf is coupled to the Brd2-mediated activation of ERK1/2. It has been reported that insulin and a PPARγ ligand (troglitazone, TZD) act synergistically to increase the expression of an adipocyte specific gene, 422/aP2 [42]. In our study we have demonstrated that Brd2 overexpression induced a decrease in 422/aP2 expression. Another mechanism by which PPARγ functions, although less well understood, is by acting synergistically with other fat regulatory factors, such as C/EBPα, to drive forward the adipogenic program [43]. Conversely, the inactivation of PPARγ could also reduce the response of these cells to C/EBPα and other fat-determining factors. Based on these findings PPARγ, might therefore be a potent target by which Brd2 signaling can promote its inhibitory effects on the development of the adipocyte phenotype. In addition, our findings indicate that Brd2 can affect the transcription of PPARγ and C/EBPα in adipocytes. Changes in PPARγ, C/EBPα and 422/aP2 expression could be the result of ERK1/2 deactivation/activation in transfected 3T3-L1 cells, and the UO126 induced PPARγ dephosphorylation, increase in PPARγ and 422/aP2 expression and restitution of the differentiation program in Brd2 overexpression cells confirm this hypothesis.

Deregulated adipogenesis can cause lipoatrophy or obesity, both of which are harmful. We have demonstrated that Brd2 may increase PPARγ phosphorylation thereby promoting degradation of PPARγ and then decreasing C/EBPα and 422/aP2 expression via an activation of the ERK1/2 pathway that suppresses adipogenesis of 3T3-L1 preadipocytes. On the other hand, disruption of Brd2 causes severe obesity in mice, although without Type 2 diabetes, however the obesity itself is a precipitating factor of many diseases, such as cancer. Therefore it seems that the maintenance of the normal Brd2 expression *in vivo* is important for body health. Recently, a small-molecule inhibitor of BET bromodomains, JQ1, with high affinity for the first bromodomain of Brd4, has received much attention for its therapeutic potential against multiple myeloma and other cancer types that are addicted to the c-Myc oncogene [44-46]. The Erk1/2 signaling pathway can be activated in response to a diverse range of extracellular stimuli including mitogens, growth factors, and cytokines [47-49], and it has been considered as an important target in the diagnosis and treatment of cancer [50]. As c-Myc has close relationship with MEK/ERK signaling [51,52], we suggest that Brd2 and its associated protein complexes should be considered in cases of obesity-associated cancer.

## Materials and Methods

### 1: Cell culture and induction of differentiation in 3T3-L1 cells

The confluent 3T3-L1 (ATCC, Manassas, VA, USA) cells were treated with 0.5 mM methylisobutylxanthane (MIX), 1 μM dexamethasone (Dex) and 1.67 μM insulin (MDI; Sigma-Aldrich, MO, USA) in Dulbecco’s modified essential medium (DMEM) containing 10% FBS for 2 days. Then, cells were transferred to DMEM with 1.67 μM insulin and 10% FBS for 2 days. Afterwards, cells were cultured in DMEM containing 10% FBS and the medium was changed every other day. Expression of adipocyte genes and acquisition of adipocyte phenotypes started on day 4 and reached a maximum by day 8. For experiments performed with EGF, cells were starved in serum free DMEM for 4 hours, and stimulated with 20 nM EGF (Cell Signaling Technology, MA, USA) for 5 min.

### 2: Vectors and transfections

A Brd2 siRNA expression vector (pSiBrd2), control (pSiC), the long-form Brd2 expression vector (pmBrd2) and an empty vector (pcDNA3.1) were gifts from Dr. F.N. Wang. A Brd2 siRNA expression vector, pSiBrd2, was constructed using pRNA-U6.1/Neo (GenScript, NJ, USA), according to the manufacturer’s protocol. The sense sequence was 5’-AGATGGGGCAGGAAGGCTCCG-3’. The annealed synthesized double-stranded fragment was cloned into the BamHI and HindIII sites of the vector. pRNAU6.1/Neo/CTL (GeneScript, NJ, USA) was used as a negative control (pSiC) for Brd2 shRNA. To construct pmBrd2, the long-form Brd2 expression vector, mouse Brd2 cDNA was obtained from R1 murine ES cells (ATCC, Manassas, VA, USA) that express a high level of *Brd2* by RT–PCR using the primers: 5’- GTGGTCGGTACCATGGTGCAAAACGTGACTCCCCACA-3’and 5’GGCTAGGAATTCAATCGTATTTTGTCCATG-3’. The cDNA was then ligated into KpnI and EcoRI sites of pcDNA3.1 (+). The empty pcDNA3.1 vector was used as a negative control for Brd2 overexpression. Briefly, 3T3-L1 adipocytes transfections were performed using Fugene 6 reagent (Roche Applied Science, Indianapolis, USA) following the manufacturer’s instructions. Cells were passaged 48 h after transfection, and treated with selection of antibiotics G418 (Sigma-Aldrich, MO, USA) for stable transfection. Single colonies were expanded.

### 3: Oil Red O staining

Cells were washed three times with PBS and then fixed for 2 min with 3.7% formaldehyde. Oil Red O (Sigma-Aldrich, MO, USA) (0.5% in isopropanol) was diluted with water (3:2), filtered through a 0.45-µm filter, and incubated with the fixed cells for 1 h at room temperature. Cells were washed with water, and the stained fat droplets in the cells were visualized by light microscopy. Oil Red O was extracted with isopropanol, and the absorbance reading was performed at 492 nm.

### 4: PCR analysis

Total RNA was isolated using the TRIzol reagent (Invitrogen, MN, USA) according to the manufacturer’s instructions. cDNA was synthesized using Superscript II reverse transcriptase (Invitrogen, MN, USA). Reverse transcription PCR was performed using DreamTaq PCR Master Mix (Thermo Fisher Scientific Inc, Waltham, MA, USA) on a PCR instrument (Eppendorf, Hamburg, Germany). Real-time PCR was performed using 2× PCR Master Mix (Power SYBR Green; Applied Biosystems, Foster City, CA) on an ABI 7300 real-time PCR instrument (Applied Biosystems, Foster City, CA). β-actin was used as the reference gene. Amplification was performed in a 20-μl reaction volume according to the manufacturer’s instructions. The primers used were as follows:

PPARγ (UniSTS:271630): 5’-CAAAGGCATGGGGTCACTT-3’ (sense) 5’-GGACAGCATATCCCTAACTTTCT-3’ (antisense);

C/EBPα (UniSTS:124964): 5’-GGTGCGTCTAAGATGAGGGA-3’ (sense)

5’-CCCCCTACTCGGTAGGAAAA-3’ (antisense)

Brd2[27]:5’-GTGGTCGGTACCATGGTGCAAAACGTGACTCCCCACA-3’ (sense) 5’-GGCTAGGAATTCAATCGTATTTTGTCCATG-3’ (antisense)

β-actin (UniSTS:270598): 5’-TGTTACCAACTGGGACGACA-3’ (sense) 5’-CTTTTCACGGTTGGCCTTAG-3’ (antisense)

### 5: Western blot analysis

Cells were washed twice with pre-chilled PBS and lysed in RIPA buffer with protease inhibitor cocktail, PMSF, and sodium orthovanadate (Roche Applied Science, Indianapolis, USA). Equal amounts of protein were separated by SDS-PAGE and transferred to polyvinylidene fluoride membranes (Millipore, Billerica, USA), immunoblotted with specific antibodies, including rabbit antibodies to phospho-PPARγ at Ser112 (Cat No.: sc-28001-R), PPARγ (Cat No.: sc-7196) and 422/aP2 (Cat No.: sc-271529) which were obtained from Santa Cruz Biotechnology (Santa Cruz, CA, USA) and rabbit antibodies to C/EBPα (Cat No.: #2295), phospho-42/44 MAPK (ERK1/2, Cat No.: #4376) at Thr202/Tyr204, ERK (Cat No.: #4695), phosphop-JNK at Thr183/Tyr185 (Cat No.: #4671), JNK (Cat No.: #9258), phospho-p38 MAPK at Thr180/Tyr182 (Cat No.: #4511), p38 MAPK (Cat No.: #8690), Raf Family Antibody Sampler Kit (Cat No.: #2330), Brd2 (Cat No.: #5848) and β-actin were obtained from Cell Signaling Technology (Cell Signaling Technology, MA, USA).

### 6: Statistical analysis

All values are shown as means ± SEM (SPSS 13.0). Paired Student’s t-tests were used to compare means, and *P* < 0.05 was considered significant.

## Supporting Information

Figure S1 **Kinetics of Brd2 expression in 3T3-L1 preadipocytes during adipogenesis.** Adipogenic differentiation of 3T3-L1 adipocytes from preadipocytes was performed; total RNA was isolated and Brd2 mRNA expression was analyzed by reverse transcription PCR.
(TIF)
