# Supplementary figures and images for: Brd2 Inhibits Adipogenesis via the ERK1/2 Signaling Pathway in 3T3-L1 Adipocytes
Source: PLoS One. 2013 Oct 23;8(10):e78536. doi: 10.1371/journal.pone.0078536 (PMC3806839; doi:10.1371/journal.pone.0078536)

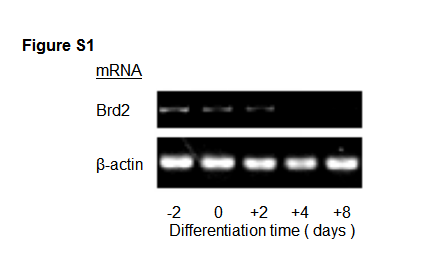

Supplement: Figure S1 — Kinetics of Brd2 expression in 3T3-L1 preadipocytes during adipogenesis. Adipogenic differentiation of 3T3-L1 adipocytes from preadipocytes was performed; total RNA was isolated and Brd2 mRNA expression was analyzed by reverse transcription PCR. (TIF) [file pone.0078536.s001.tif]
